# Drug-Coated Balloon for the Treatment of Nonacute Symptomatic Intracranial Carotid Artery Terminus Occlusion: Initial Experience and Follow-Up Outcome

**DOI:** 10.3389/fneur.2022.840865

**Published:** 2022-02-11

**Authors:** Hao Yin, Jinping Zhang, Wei Zhao, Meimei Zheng, Yun Song, Lili Sun, Jun Zhang, Ju Han

**Affiliations:** Department of Neurology, The First Affiliated Hospital of Shandong First Medical University, Jinan, China

**Keywords:** drug-coated balloon, recanalization, nonacute symptomatic, intracranial carotid artery terminus, occlusion, endovascular treatment

## Abstract

**Background:**

Studies on the recanalization for occlusion of the internal carotid artery terminus are scattered. Recently, drug-coated balloon (DCB) has been increasingly applied in the intracranial artery occlusion and achieved encouraging results. However, there seems no convincing data for the nonacute symptomatic internal carotid artery terminus occlusion (sICATO).

**Objective:**

To assess the feasibility and effectiveness (safety) of DCB for patients with nonacute sICATO refractory to medical therapy.

**Approach:**

This study included 30 patients with nonacute sICATO treated with DCBs and/or remedial stenting. The rate of successful recanalization, periprocedural complications, and clinical and vascular imaging follow-up outcomes were retrospectively analyzed.

**Results:**

Drug-coated balloon (DCB) dilatation of nonacute sICATO gives a 100% rate of successful recanalization, with a low complication rate (10.00%), good clinical outcomes (86.20%), low restenosis/reocclusion rate (3.45%), and one asymptomatic ipsilateral infarction (3.45%).

**Conclusion:**

Drug-coated balloon dilation seems to be the promising treatment option for nonacute sICATO considering its safety and feasibility.

## Introduction

Intracranial atherosclerosis (ICAS) is an important cause of stroke leading to permanent damage to the brain, especially among Asian populations. Nonacute internal carotid artery occlusion (ICAO) may cause fluctuating clinical symptoms, namely, minor/major stroke or recurrent transient ischemic attack, or asymptomatic (1). The reason is that the blood supply reduction to the perfused territory is usually compensated by intracranial and extracranial–intracranial collaterals. Symptomatic cerebral ischemia associated with ipsilateral ICAO occlusion accounts for 5–8% of a recurrent ischemic stroke every year (2, 3). In addition, it is reported that among nonacute ICAO patients with compromised hemodynamic status, recurrent stroke risk increases to ~12% per year (4), even as high as 86% in a 7-year follow-up study (5).

Endovascular treatment is generally considered for nonacute ICAO if medical management, namely, dual-antiplatelet treatment (DAPT), statin, risk factor management, and lifestyle interventions fail or as a prophylaxis treatment for high-risk patients (6). Indication for endovascular treatment and the techniques used were not standardized. It is recommended among symptomatic patients with stage I or II hemodynamic failure (7).

A meta-analysis (8) reported endovascular treatment of nonacute ICAO limited to the cervical internal carotid artery (ICA) is feasible, with a 70% rate of successful recanalization. There were a few case-series studies that have reported stenting appeared to be safe and efficient for nonacute intracranial artery occlusion, including intracranial ICATO (9–11). Previous studies showed that the technical success rates of stenting for intracranial ICAO were high (10, 11). However, the reopened segment would be more prone to restenosis/reocclusion even after successful recanalization (9, 12, 13). The recanalization of the restenosis/reocclusion would be more difficult due to the stent.

Accordingly, there would need to be a viable alternative for nonacute ICAO recanalization. DCB, the coated balloon with antiproliferative drugs, paclitaxel, can effectively inhibit smooth muscle cells proliferation and migration by irreversibly stabilizing intracellular microtubules, therefore reducing the risk of restenosis compared with conventional balloon dilatation and stenting (14, 15). It is off-label used in the intracranial artery. Our previous experiences have shown the safety and feasibility of DCB for intracranial *de novo* atherosclerosis disease, with a lower incidence of restenosis (16, 17).

In this single-center study, we aimed to assess the feasibility and the safety of the endovascular treatment and to investigate if selected patients may benefit from DCB treatment when medical therapy failed in patients with nonacute sICATO underlying ICAS.

## Materials and Methods

### Study Population

In this retrospective study, we recruited consecutive patients diagnosed with nonacute sICATO by digital subtraction angiography (DSA) in The First Affiliated Hospital of Shandong First Medical University between January 2016 and October 2020.

Intracranial atherosclerosis was the primary etiology for all target arteries. All patients had the instable clinical syndrome, such as recurrent transient ischemic attack (TIA), stroke, and neurologic deterioration (progressive or crescendo stroke). They did not achieve satisfactory improvement despite the best medical therapy (BMT), namely, DAPT, statin, blood pressure augmentation therapy, optimized glucose control, smoking cessation, and other lifestyle interventions. The occlusion length of type I lesions was <10 mm extending from the ophthalmic artery segment to the proximal anterior communicating artery. These lesions were detected by computed tomographic angiography (CTA), magnetic resonance angiography (MRA), and confirmed by DSA. Although DSA showed favorable patency of distal vasculature and collateral circulation, MRI depicted infarctions located at the cortical or subcortical borderzone territory and small infarction core shown on diffusion-weighted imaging (DWI) with a large area of low perfusion of ICA territory assessed by arterial spin labeling (ASL).

In this study, other potential causes of occlusion, such as vasculitis, Moyamoya syndrome, emboligenic heart, or arterial dissection were not included. Patients who had no recurrent ischemic events after BMT were not included. If the collateral circulation between the anterior cerebral artery and/or posterior cerebral artery was well-developed and perfusion of ICA territory assessed by DSA was adequate, patients did not need the procedure.

All the enrolled patients or their authorized surrogates knew the risks and benefits of endovascular treatment, including the off-label use of the coronary DCB and gave a written informed consent in accordance with the Declaration of Helsinki. The institutional review board of The First Affiliated Hospital of Shandong First Medical University approved the study without registration owing to its retrospective nature.

### Preprocedural Medical Management

A DAPT with 100 mg aspirin and 75 mg clopidogrel was routinely maintained for at least 5 days before the procedure. Thromboelastography platelet mapping was tested to assess bleeding risk and guide perioperative DAPT. If drug inhibition occurs, the effectiveness of the drug can be guaranteed by increasing the dosage or changing the drug, such as ticagrelor. If the bleeding risk elevates, decreasing the drug dosage can adjust the effectiveness. All patients were given individual standard medical treatment and lifestyle interventions, namely, antihypertensive agents, antidiabetic drugs, and statins.

### Intervention Procedure

All the procedures were performed *via* the percutaneous transfemoral route under general anesthetic. Heparin was administered intravenously to keep the activated clotting time between 250 and 300 s during the procedure.

A 6F 90-cm-long sheath and Catalyst 058 115 cm intermediate catheter coaxially advanced until catalyst reached the proximal to the ophthalmic artery. A 0.014 Synchro microguide wire (Stryker Neurovascular, Salt Lake City, Utah, USA) was used to enter the occluded internal carotid terminus with the support of an Excelsior SL-10 soft microcatheter (Stryker Neurovascular, Cork, Ireland). Once we cross the proximal occlusion, the microcatheter was advanced until we reach the patent lumen. Through the microcatheter angiography, the length of the occlusion and distal lumen of the lesion were confirmed after withdrawing the microguidewire. Once it is confirmed, the microguide wire was advanced through the microcatheter and positioned in the M2 segment. We then use the microguide wire as a railroad for delivering different devices to reconstruct the occluded segment from distal to proximal. The lesions were initially inflated with conventional balloons (Gateway balloon, Boston Scientific, Maple Grove, Minnesota, USA). The subsequent application of paclitaxel-coated coronary balloon (SeQuent Please, B. Braun, Berlin, Germany) and/or stent depended on the decision of the operator. The application method of the DCB was the same as described in our previous study (16). The intervention was considered a technical success if the occlusion segment was processed with establishing grade 2b-3 antegrade thrombolysis in cerebral ischemia (TICI) flow. Residual stenosis is defined as > 50% stenosis at the end of the intervention.

### Postprocedural Management

After the procedure, a cerebral CT scan was performed immediately. And all patients were intensively monitored including keeping the systolic blood pressure ≤ 130 mmHg and documenting neurological symptoms/signs. Assessment of the National Institutes of Health Stroke Scale (NIHSS) score was performed by the neurologist within 24 h after the procedure. The investigated complications included distal embolization, intracranial hemorrhage, hyperperfusion syndrome, dissection, and death.

### Follow-Up Management and Data Collection

Aspirin 100 mg with clopidogrel 75 mg/day or ticagrelor 90 mg two times 1 day was maintained for 3 months for patients with only DCB dilatation, 6 months for patients with remedial stenting implantation. Then the dual antiplatelet regimen was transitioned to a single antiplatelet (aspirin or clopidogrel or ticagrelor) therapy to be continued for the lifetime of patients.

We collected the demographic, clinical, angiographic, and procedural data. All patients underwent clinical follow-up at 1 month, 3 months, 6 months, and 1 year to evaluate the functional outcome and rates of recurrent TIA, stroke, and death. The NIHSS and the modified Rankin Scale (mRS) were used to, respectively, access the severity of the clinical stroke and functional outcome at admission, preprocedure, 24 h postprocedure, at discharge, 30-day postprocedure, and every follow-up. The mRS is commonly applied to assess the degree of disability in patients suffering a neurological event, ranging from 0 (asymptomatic) to 6 (death) in our study (18). A favorable functional outcome was defined as the mRS score ≤2 at 3 months.

All the patients were scheduled to reperform vascular imaging examination at 3 months for patients with only DCB dilatation, 6 months for patients with remedial stent implantation. DSA was preferred, but MRA/CTA was also accepted for some patients who refuse to perform DSA. Angiographic or in-stent restenosis (ISR) was defined as, within or immediately adjacent (within 5 mm) of the treated segment, a diameter of the stenosis >50% for Preoperative residual stenosis ≤30 or >20% absolute luminal loss for Preoperative residual stenosis >30%. Angiographic or in-stent reocclusion was defined as the recanalized lumen being completely lost. Consensus resolved disagreements. The restenosis/reocclusion, associated with ischemic symptoms of the offending vessel territory, is called symptomatic restenosis/reocclusion.

### Statistical Analysis

Descriptive statistics were used in this study. Continuous data were expressed as the mean ± SD or as the median with interquartile range (IQR). It was compared by using the Student's *t*-test or the Mann–Whitney *U*-test. Categorical data were expressed as numbers and percentages, and compared using the chi-squared or Fisher's exact test. Statistical analysis was performed using SPSS version 23.0 (SPSS Inc., Chicago, IL, USA).

## Results

### Characteristics of Patients

A total of 30 patients, 16 males and 14 females (53.33%, 46.67%), were enrolled in this study. The baseline demographic and clinical characteristics of the 30 patients are given in Table 1. The mean age of the enrolled patients was 57.27 ± 10.12 years, with no gender differences. The primary medical history of these patients included hypertension (20, 66.67%), diabetes mellitus (13, 43.33%), hyperhomocysteine (10, 33.33%), cardiovascular disease (6, 20.00%), hyperlipidemia (5, 16.67%), and atrial fibrillation (2, 6.67%). Hypertension (*N* = 20, 66.67%), diabetes mellitus (*N* = 13, 43.33%), and smoking (12, 40.00%) were the most common risk factors. The median NIHSS and the mRS scores at baseline were 3.0 (0–7.5) and 2.0 (IQR, 1–4), respectively.

**Table 1:** Baseline demographic and clinical characteristics of patients.

**Characteristics**	***n* = 30**
**Demographics**	
Sex, male	16 (53.33%)
Female	14 (46.67%)
Age, years, mean ± SD	57.27 ± 10.12
**Medical history**	
Hypertension	20 (66.67%)
Diabetes mellitus	13 (43.33%)
Hyperhomocysteine	10 (33.33%)
Cardiovascular disease	6 (20.00%)
Hyperlipidaemia	5 (16.67%)
Atrial fifibrillation	2 (6.67%)
Smoking	12 (40.00%)
**Clinical**	
mRS, median (IQR)	2.0 (1–4)
NIHSS, median (IQR)	3.0 (0–7.5)
Preoperative NIHSS, median (IQR)	3.0 (0–9.25)

*mRS, modified Rankin Scale; NIHSS, National Institutes of Health Stroke Scale; IQR, interquartile range*.

### Preoperative MRI and Angiographic Findings

**Figure 1:**
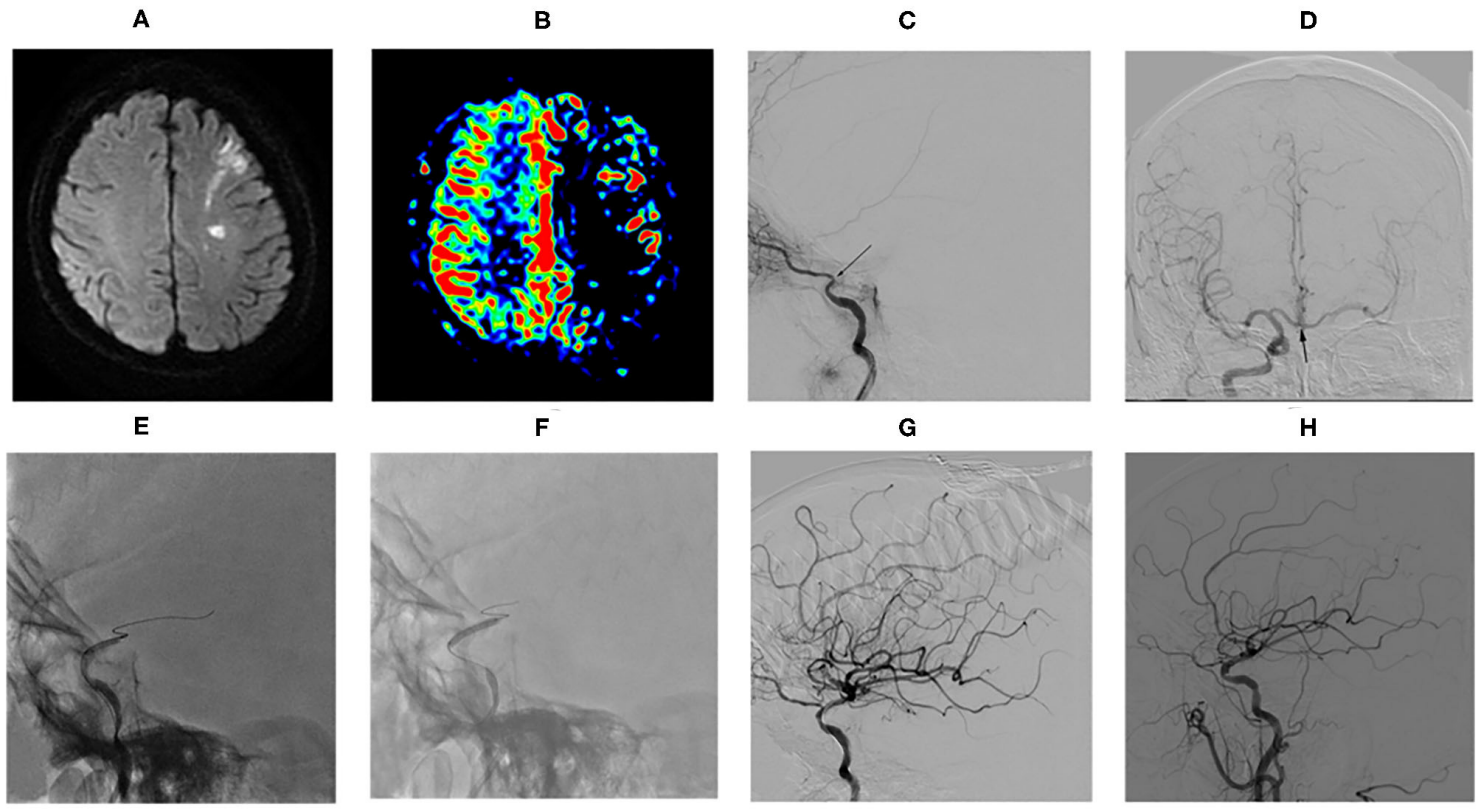
Example of drug-coated balloon (DCB) dilatation for nonacute sICATO and follow-up. **(A)** MRI revealed left watershed infarction. **(B)** ASL showed left cerebral hemispheric hypoperfusion in the ICAO territory. **(C)** DSA confirmed the left internal carotid artery terminus occlusion ipilaterally to the infarcts (arrow). **(D)** DSA showed anterior communicating arteries (arrow). **(E)** Predilatation with a conventional balloon. **(F)** DCB dilatation after predilatation. **(G)** Angiographic result after the procedure. **(H)** Angiographic result at follow-up of 3.0 months.

Magnetic resonance imaging results revealed acute infarcts in the cerebral hemisphere (involving the frontal lobe, temporal lobe, parietal lobe, occipital lobe, semiovale center, basal ganglia area, and corona radiata area), usually presenting as a watershed infarction (Figure 1A). ASL assessed complete or partial hypoperfusion in the ICAO territory (Figure 1B). CTA/MRA detected the internal carotid artery terminus occlusion (ophthalmic segment, C6 and/or communicating segment, C7) ipsilaterally to the infarcts. The above were confirmed by DSA (Figure 1C). In addition, DSA showed all patients had collateral circulation, at least including one of the primary collaterals (anterior and/or posterior communicating arteries, AcomA and/or PcomA) (Figure 1D) and the secondary collaterals (leptomeningeal and/or ophthalmic arteries LC and/or OAs).

### Procedural Characteristics

Overall, the median time from the symptom onset to treatment and the occlusion confirmed to treatment was 29 days (range, 6.0–270.0 days; IQR, 20.0–67.5 days) and 26 days (range, 7.0–300.0 days; IQR, 18.0–75.0 days), respectively. All patients achieved stable antegrade perfusion with TICI 3 in 16 cases (16/30, 53.33%) and TICI 2b in 14 cases (14/30, 46.67%). Among the 30 successful patients, DCB angioplasty (Figures 1E–H) was only applied in 22 patients, while DCB angioplasty plus remedial stenting was applied in 8 patients. The postprocedure stenosis was 14.0 ± 21.1%. The residual stenosis rate was 6.67% (2/30). A total of 4 cases had no residual stenosis but the lumen wall was irregular because of neointima formation, vascular remodeling, or plaque. Asymptomatic vessel dissection after DCB inflation occurred in 1 patient (3.33%). No obvious periprocedural complications and hemorrhage happened. Remedial stenting was not performed due to table antegrade perfusion with TICI 2b in this patient. Periprocedural distal embolization strokes occurred in 2 patients (6.67%). They had blurred vision on the ipsilateral to the recanalized vascular. Other common periprocedural complications, such as cerebral hemorrhage, hyperperfusion syndrome, or death did not occur in this case series. The treatment modalities and outcomes of the 30 patients are summarized in Table 2.

**Table 2:** Angiographic and procedural characteristics.

**Characteristics**	***n* = 30**
**Timing**	
Symptom onset to treatment, days, median (IQR)	29 (20.0–67.5)
Occlusion confirmed to treatment, days, median (IQR)	26 (18.0–75.0)
**Technical success** ^**a**^	30 (100%)
**Modality of recanalization**	
Angioplasty with DCB	22 (73.33%)
Angioplasty with DCB and remedial stenting	8 (26.67%)
**Postprocedural Perfusion**	
TICI = 2b	14 (46.67%)
TICI = 3	16 (53.33%)
**Residual stenosis** ^**b**^	2 (6.67%)
**Complication rate**	
Distal embolization	2 (6.67%)
Dissection	1 (3.33%)
Hyperperfusion syndrome	0
ICH	0
Death	0

a *Technical success, defined as TICI ≥ 2b at the end of the intervention*.

b *Residual stenosis, defined as > 50% stenosis at the end of the intervention*.

*DCB, drug-coated balloon; TICI, thrombolysis in cerebral ischemia; ICH, intracranial hemorrhage*.

### Clinical and Angiographic Outcomes

One patient was lost during the follow-up. The rest of 29 patients (96.67%) all received clinical follow-up with no death, and 21 of them (70.00%) were followed up radiologically. During the clinical follow-up period of 7.02 ± 3.65 months, a new asymptomatic ipsilateral infarction based on MRI occurred in 1 patient at 30 days (3.45%). After 3 months, this patient herself transited a dual antiplatelet regimen to a single antiplatelet (aspirin) therapy without any further vascular imaging examination. The patient who had symptomatic vessel dissection experienced the asymptomatic reocclusion of the recanalized R-ICA terminus at 30-day angiographic follow-up. After 4 months, this patient developed paroxysmal dizziness and numbness in the left limb with DWI detecting no new ischemic lesion.

**Figure 2:**
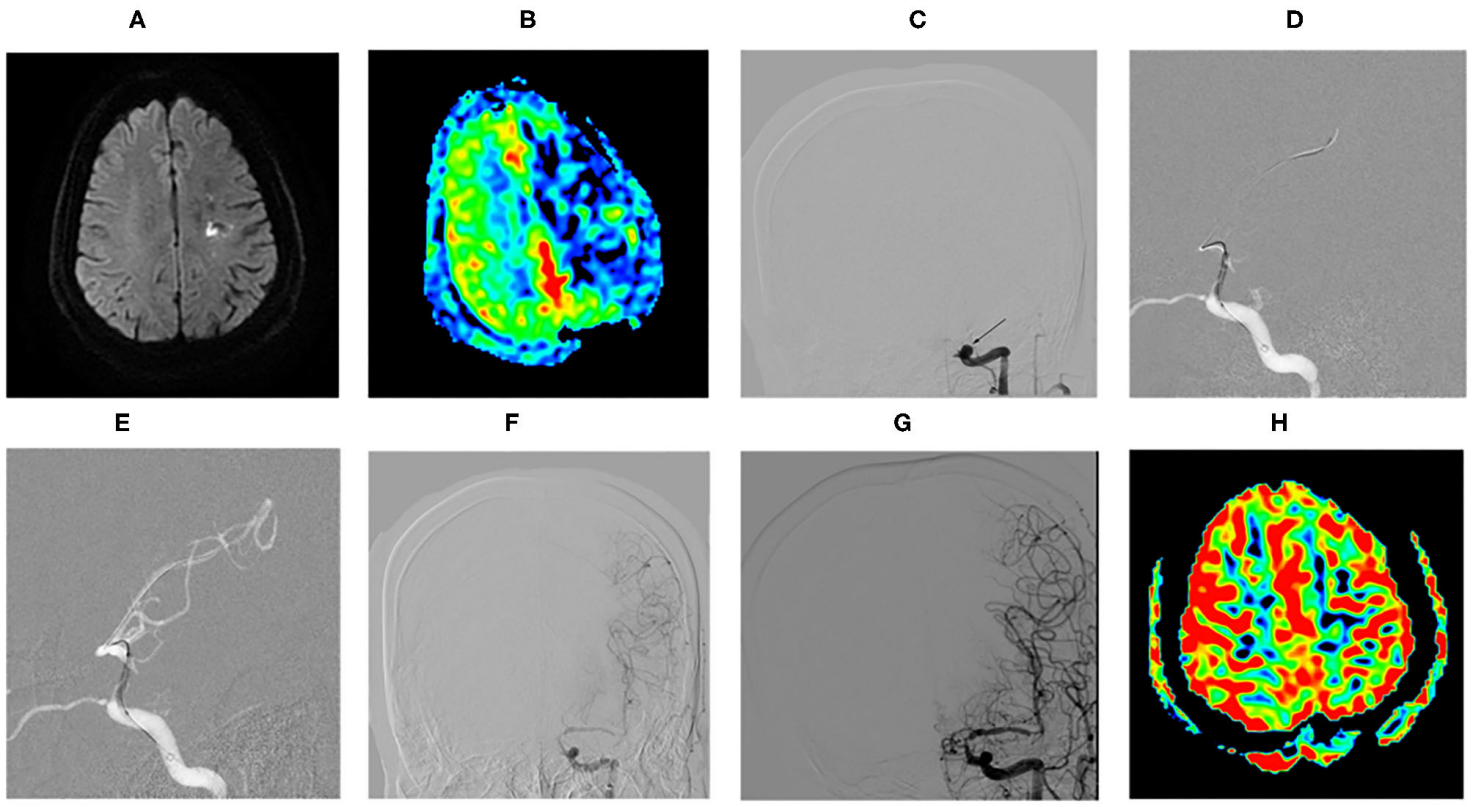
Example of comparing the preoperative MRI and operative angiographic findings with follow-up. **(A)** MRI revealed left watershed infarction. **(B)** ASL showed left cerebral hemispheric hypoperfusion in the ICAO territory. **(C)** DSA confirmed the left internal carotid artery terminus occlusion ipilaterally to the infarcts (arrow). **(D)** Predilatation with a conventional balloon. **(E)** DCB dilatation after predilatation. **(F)** DSA showed the occlusion recanalization with 70% residual stenosis with a large number of thrombus (arrow). **(G)** DSA showed thrombus disappeared and residual stenosis rate improved with 50% residual stenosis at angiographic follow-up of 1 month (arrow). **(H)** ASL showed basically symmetric bilateral cerebral hemispheric perfusion at angiographic follow-up of 1 month.

The proportion of patients with a good clinical outcome (mRS score 0–2) was 86.20% (25/29), and 89.66% (26/29) achieved an acceptable outcome (mRS score 0–3). A total of 3 patients received mRS score of 4 at the final clinical follow-up. There was a significant difference between the follow-up mRS and the Preoperative mRS (*p* = 0.00, < 0.05). During the vessel imaging examination at 8.02 ± 3.65 months, cerebral angiography was obtained for 23 patients (76.67%). DSA follow-up and MRA/CTA follow-up were available for 16 patients and 7 patients, respectively. No restenosis/ISR occurred. There was no significant difference between the follow-up and the postoperative residual stenosis (*p* = 0.072, >0.05). Residual stenosis degree in 2 cases was reduced to ≦50% at follow-up. As shown in Figure 2, this patient who was found a large number of thrombus during the occlusion recanalization with 70% residual stenosis, reexamined DSA showing thrombus disappeared and residual stenosis rate improved with 50% residual stenosis at 30-day angiographic follow-up. Another patient was found residual stenosis rate reducing from 60 to 40%. There was no significant difference between the follow-up and the postoperative TICI grade (*p* = 0.136, >0.05). But among 9 patients who achieved postprocedural stable antegrade perfusion with TICI 2b, 7 patients received the significantly improved TICI grade from 2b to 3 (77.78%) at angiographic follow-up.

## Discussion

In this single-center retrospective pilot study, we found that DCB angioplasty was feasible and safe in the treatment of patients with nonacute sICATO.

Nonacute progression of occlusive lesions may permit distal collateral development to avert major ischemic events. The primary collaterals include ACoM or PCoM and secondary, LC or OA. The availability of collateral supply tends to curb the potential ischemic injury. The patients suffering from nonacute ICAO are usually asymptomatic or cause fluctuating clinical symptoms, including recurrent transient ischemic attack or minor/major stroke (1). Until collateral circulation cannot compensate for the reduced downstream perfusion or emboli fall from the stump of the ICA *via* collaterals (19–21), ischemic stroke symptoms appear with radiography depicting infarcts in the cortical or subcortical borderzone territory (anterior borderzone between ACA and MCA territories and posterior borderzone between MCA and PCA territories) ipsilateral to the occlusion. The internal carotid artery terminus is located between the ophthalmic artery segment and the proximal anterior communicating artery with the opening of the PcomA or AcomA. It is a relatively short occlusion (≤10 mm). The location of the vascular occlusion is important because of the good reconstruction of distal collateral vessels from the PcomA or AcomA, in that ICA occlusions were significantly correlated with both mortality and good clinical outcome. Our patients having recurrent cerebral ischemic symptoms despite BMT with favorable collateral circulation shown by DSA were chosen to recanalize the occlusive ICA. Our results suggest that patients with an occlusion of the ICA terminus had a high rate of successful recanalization, low periprocedural major complications, and better follow-up patency rates. This finding is consistent with previous research (22).

The incidence of ISR increases the risk of recurrent ischemic events. Symptomatic ISR was ~9.6–14.0% (23–25). A high ISR rate is a challenge for intracranial stenting. DCB could effectively reduce the risk of restenosis/reocclusion. Patients are allowed to receive noninvasive magnetic resonance examination because of no mechanical scaffold after DCB angioplasty, which reduces the technical difficulty of treating re-events. SeQuent Please DCB was initially designed for coronary arteries. For intracranial arteries, the DCB might be not a suitable size and its tip might be somewhat rigid. Meanwhile, an excessively tortuous vessel pathway increases the difficulty and risk of the procedure. Therefore, we strictly evaluated the vessel path to select the proper patient. During the procedure, an intracranial support catheter helps navigate to the target artery, which might have accounted for the higher arrival rate of DCBs compared with a previous report (26). In addition, for the drug working on the blood vessel walls better, it is important to use a bare balloon for the lesion predilation before DCB inflation.

In this study, only 2 patients (2/30, 6.67%) had blurred vision on ipsilateral to the recanalized vascular owing to the distal embolization. It is similar to our initial experience (16) and some small sample studies (27, 28). Re-events occurred rate is obviously low about 3.35% (1/29) in this study, which is according to our initial experience (16) and further research (17). The patient who had dissection with stable antegrade perfusion after the operation did not perform stent. This patient experienced the reocclusion of the recanalized R-ICA terminus at a 30-day angiographic follow-up. Therefore, further study will investigate the timing of remedial stenting and compare the re-events occurred rate between DCB dilation with and without a remedial stent.

Although there was no statistically significant difference in either residual stenosis or TICI grade, the residual stenosis, and irregular vascular walls, were improved, even became regular vascular walls at angiographic follow-up. Therefore, our study showed that the role of DCB seems to be just as important in vascular remodeling and patency improvement for intracranial arteries as for coronary artery disease (25).

In this study, all the 30 arteries achieved good lumen patency and antegrade flow. A total of 8 patients underwent remedial stent implantation, usually self-expanding stent or balloon-mounted stent, due to unsustainable effective antegrade flow, and 22 patients recanalized with DCB only. Except for 1 patient lost during follow-up, 26 of the rest 29 patients achieved acceptable clinical follow-up outcomes. Compared with preprocedure, most patients showed statistically significant improvement at mRS score at follow-up. The reason for mRS grade 4 seems to be massive cerebral infarction (1/30) or progressive stroke (2/30) leading to severe neurological impairment. Even the occlusive artery recanalized and postoperative rehabilitation exercise continued, but it did not work.

### Limitations

There are several limitations in this study. First, the mean age of the patients was 57 years, a little younger than the average patients with ICAS, there might be a selection bias. It might be because this study is of a retrospective nature and monocentric design with not large enough sample size. In the future, randomized controlled trials are needed to eliminate the age bias and investigate whether this treatment compares favorably with BMT and non-DCB therapy in these patients. Second, MRA and CTA follow-up were performed in some patients so that detail may partly jeopardize the consistency. Third, the angiographic follow-up time was scheduled for <1 year, the efficacy of the procedure needs to be re-evaluated during a long-term follow-up. Therefore, we need to develop a more reasonable follow-up plan and strengthen follow-up management.

### Interpretation

To the best of our knowledge, this is the first case series of patients with nonacute sICATO treated with DCB. We systematically reviewed the safety and efficacy of DCB. DCB-oriented angioplasty might be considered a viable alternative treatment for patients with nonacute sICATO who failed standard medical treatment.

## Conclusion

This series study shows that DCB may be feasible and safe for nonacute atherosclerotic sICATO disease with recurrent stroke attributed to impaired cerebral hemodynamics refractory to medication in carefully selected patients. It should be emphasized that revascularization of nonacute sICATO is a high-risk procedure; therefore, the selection of eligible patients and perfect treatment of complications are equally critical. Further prospective randomized studies with larger patient numbers and longer follow-up periods are mandatory to investigate the clinical efficacy and indications of the procedure.

## Data Availability Statement

The raw data supporting the conclusions of this article will be made available by the authors, without undue reservation.

## Ethics Statement

The studies involving human participants were reviewed and approved by the Institutional Review Board of The First Affiliated Hospital of Shandong First Medical University. The patients/participants provided their written informed consent to participate in this study. Written informed consent was obtained from the individual(s) for the publication of any potentially identifiable images or data included in this article.

## Author Contributions

JH, HY, and JiZ: study concepts and design. HY and MZ: statistical analysis. HY, JiZ, JuZ, YS, and LS: manuscript composition. HY, WZ, and JH: manuscript revision. All authors contributed to the article and approved the submitted version.

## Conflict of Interest

The authors declare that the research was conducted in the absence of any commercial or financial relationships that could be construed as a potential conflict of interest.

## Publisher's Note

All claims expressed in this article are solely those of the authors and do not necessarily represent those of their affiliated organizations, or those of the publisher, the editors and the reviewers. Any product that may be evaluated in this article, or claim that may be made by its manufacturer, is not guaranteed or endorsed by the publisher.
